# Sex-specific differences in biomechanics among runners: A systematic review with meta-analysis

**DOI:** 10.3389/fphys.2022.994076

**Published:** 2022-09-23

**Authors:** Ping-Ping Xie, Bíró István, Minjun Liang

**Affiliations:** ^1^ College of Science and Technology, Ningbo University, Ningbo, China; ^2^ Faculty of Engineering, University of Szeged, Szeged, Hungary; ^3^ Faculty of Sports Science, Ningbo University, Ningbo, China

**Keywords:** running, biomechanics, physical therapy, kinematics, hip

## Abstract

Patellofemoral disorders are more common in female runners compared to their male counterparts. Differences in biomechanical characteristics between groups of runners could provide insight into the causes of higher rates of injury in female versus male runners, which would be useful to physical therapists and athletic trainers in development of individualized injury prevention programs. This review compares the differences in biomechanical characteristics between female and male runners. Electronic databases, including PubMed, Scopus, Web of Science, and Embase were searched in December 2021 for studies evaluating sex-specific differences in lower limb mechanics of healthy participants during running. Two independent reviewers determined the inclusion and quality of each research paper. Meta-analyses were used where possible. A total of 13 studies were selected. Means and standard deviations of reported data were retrieved from each selected paper for comparison of results. Three biomechanical variables, including dynamics, muscle activation, and kinematics, were compared between female and male runners. However, no differences were found in kinetic variables or muscle activation between groups due to insufficient data available from the selected studies. Meta-analyses of kinematic variables revealed that female runners exhibited significantly greater hip flexion angle, hip adduction angle, and hip internal rotation angle, but smaller knee flexion angle compared to male runners during running. We found significant differences in kinematic variables between female and male runners, which could influence the training advice of physical therapists and athletic trainers who work with runners, and inform the development of injury prevention programs.

## Introduction

Running is one of the most popular sports throughout the world. The idea that “exercise is medicine” is widely publicized and running is strongly recommended for the prevention and rehabilitation of many health issues (Pedersen and Saltin, 2015; Pedisic et al., 2020; Dempster et al., 2021). There are currently more than 30 million regular runners in the United States and Europe, with 36% considered recreational runners (Almeida et al., 2015). Although running is regarded as a simple activity, it coordinates complex motor integration in which all body segments and joints work in unison. Unfortunately, running-related injuries are common and associated with high prevalence of injury in the lower extremities, with rates ranging between 18 and 92% (Almeida et al., 2020). The most frequent running-related injuries are ankle sprain, patellofemoral pain syndrome, and Achilles tendinopathy (Fiorese et al., 2020).

Special attention has been given to sex-specific differences in running biomechanics and running-related injuries. Female runners are reported to be two-fold more likely to sustain certain running-related injuries compared to their male counterparts, with the incidence of running-related injury between 62–76% in female and 24–32% in male runners (Taunton et al., 2002). The sex-specific morphology of bony structure may contribute to differences in running-related injuries, as female runners have a larger hip-width to femoral length ratio compared to male runners (Ferber, Davis, and Williams Iii, 2003). Sex-specific differences in the morphology of the pelvis and thigh can lead to variation in biomechanical characteristics. In addition, abnormal mechanics are thought to be an important contributing factor in running-related injury. In comparison with male runners, female runners present greater frontal and transverse planes of motion in the lower extremities during running. Specifically, female runners exhibit greater hip internal rotation and adduction, as well as greater peak knee abduction, compared to male runners (Malinzak et al., 2001; Ferber, Davis, and Williams Iii, 2003). It has been suggested that the increased frontal and transverse planes of motion of the lower limbs lead to various running-related injuries, such as patellofemoral disorder and iliotibial band syndrome (Fredericson et al., 2000; Leetun et al., 2004; Nohren, Davis, and Hamill, 2007). Additionally, Ceyssens et al. (2019) performed a systematic review and concluded that the greater peak hip adduction movements in female runners were associated with increased lower limb morbidity, presenting as issues such as patellofemoral pain and iliotibial band syndrome.

Biomechanical differences in the lower limbs between male and female runners also affect running economy. It has been demonstrated that mechanical work plays a determinant role in energy expenditure during human movement (Peyré-Tartaruga and Coertjens, 2018; Peyré-Tartaruga et al., 2021). Factors such as joint kinematics, kinetics, and muscle activity significantly influence running economy. More specifically, Folland et al.(2017) found that differences in vertical oscillation of the pelvis, knee joint angle, and horizontal pelvis velocity caused by running performance could lead to substantial changes in running economy. In previous work, Daniels and Daniels (1992) revealed that male runners were more economical, demonstrating less oxygen use at a given speed, compared to female runners, and that this difference may contribute to the discrepancy in running performance between male and female runners.

Therefore, a comprehensive understanding of the differences in kinetic and kinematic patterns between male and female runners during running may help explain sex-specific rates and types of injury, and accounting for differences in biomechanical mechanisms may increase the effectiveness of injury prevention measures. To the best of our knowledge, no prior systematic review has been conducted to assess differences in running biomechanics between female and male runners. Therefore, the aim of this systematic review and meta-analysis was to explore the biomechanical differences in kinematic and kinetic variables between male and female runners.

## Methods

### Search strategy and inclusion criteria

This review was conducted according to PRISMA guidelines for presenting systemic reviews and meta-analyses (Moher et al., 2009). Two independent authors performed systematic literature searches for published articles from publication inception through December 2021. Randomized controlled trials and prospective cohort studies that involved healthy male and female runners were included. The searches were limited to English language articles published in peer-reviewed journals. Furthermore, only studies investigating biomechanical variables during running that enrolled both male and female adult participants were included. The studies explored kinematic and kinetic variables, muscle activation patterns, and spatial-temporal variables related to all joints and body segments. The running disciplines included, but were not limited to, middle and long-distance treadmill, road, trail, and cross-country running. There were no limits on the experimental measurement methods and equipment used to obtain biomechanical parameters. Systematic reviews, case studies, retrospective studies, commentaries, cross-sectional studies, and clinical trials were excluded.

To efficiently conduct the literature search, specific keywords were used in four different databases (PubMed, Scopus, Web of Science, and Embase). All databases were checked to confirm identification of relevant articles based on title, abstract, and keywords. Two independent researchers selected relevant articles according to the inclusion criteria. A third investigator was available to make a consensus decision if there was any disagreement regarding study inclusion or exclusion.

### Quality assessment of selected studies

Two independent reviewers examined the risk of bias for all retrieved articles based on the modified version assessment of the Downs and Black Quality Index, which has been used in a previous systematic review evaluating biomechanical studies (Jafarnezhadgero et al., 2021). The assessment form included 20 items, with nine items for information reporting, two items for internal validity, four items for external validity, and five items for selection bias. Each item was assigned a 0 or 1 score to represent the risk of bias in the selected studies. The assessment criteria were divided into three categories: high risk of bias (score of 0–6); moderate risk of bias (score of 7–13); low risk of bias (score of 14–20) (Appendix Table A1). Details of the risk of bias assessment of retrieved articles are shown in Table 1. If there was any disagreement, the third reviewer was consulted to reach a consensus on the risk of bias for each article.

**TABLE 1:** Quality assessment of included studies.

Ref	Items																				Score
Ferber et al. (2003)	1	2	3	4	5	6	7	8	9	10	11	12	13	14	15	16	17	18	19	20	
Takabayashi et al. (2017)	√	√	√	√	√	√	√	x	√	√	√	√	√	√	√	√	√	√	√	x	18
Almonroeder and Benson (2017)	√	√	√	√	x	√	√	x	x	√	√	√	√	√	√	√	√	√	√	x	16
Willson et al. (2012)	√	√	√	√	x	√	√	x	x	√	√	√	√	√	√	√	√	√	√	x	16
Vannatta and Kernozek (2018)	√	√	√	√	x	√	√	√	√	√	√	√	√	√	√	√	√	√	√	x	18
Sinclair et al. (2014)	√	√	√	√	√	√	√	√	x	√	√	√	√	√	√	√	√	√	√	x	18
Phinyomark et al. (2014)	√	√	√	√	x	√	√	x	√	√	√	√	√	√	√	√	√	√	√	√	18
Rye (2017)	√	√	√	√	√	√	√	x	x	√	√	√	√	√	√	√	√	√	√	x	17
Sakaguchi et al. (2014)	√	√	√	√	x	√	√	x	√	√	√	√	√	√	√	√	√	√	x	√	17
Sinclair et al. (2012)	√	√	√	√	x	√	√	√	x	√	√	√	√	√	√	√	√	√	x	√	17
Chumanov et al. (2008)	√	√	√	√	x	√	√	x	√	√	√	√	√	√	√	√	√	√	√	√	18
Hannigan et al. (2018)	√	√	√	√	√	√	√	x	x	√	√	√	√	√	√	√	√	√	√	x	17
Schache et al. (2003)	√	√	√	√	x	√	√	x	x	√	√	√	√	√	√	√	√	√	√	x	16

### Data extraction and analysis

Study characteristics, such as the sample size, participant characteristics, evaluation methodology, and outcome parameters were included. Data extraction from relevant papers was conducted by one independent investigator. The biomechanical parameters from selected studies were classified into four categories: kinematic, kinetic, muscle activity, and spatial-temporal. Corresponding authors of the selected articles were contacted by email to ask for missing data when necessary.

To compare biomechanical variables between male and female runners, a meta-analysis via a random-effects model was used to calculate standard mean differences for all values (*p* < 0.05). Mean and standard deviation values were presented for outcomes when these data were available from at least three studies that assessed the same outcomes using comparable methodology (e.g., sex-specific biomechanical differences during running).

## Results

**FIGURE 1:**
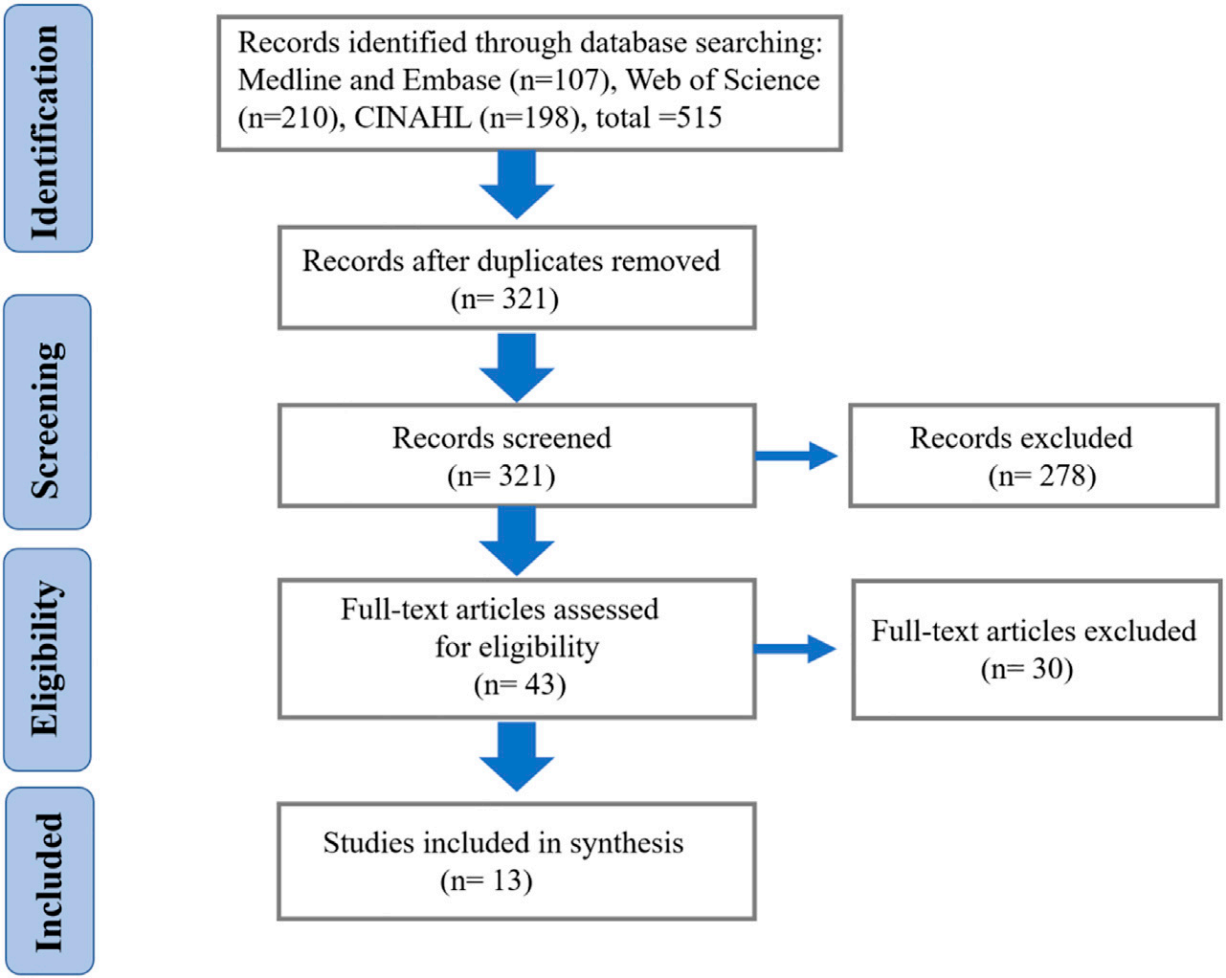
The process of article selection.

Our literature search resulted in a total of 515 citations. After selection based on our inclusion criteria, 10 articles were included in the systematic review. The systematic search process and screening details are shown in Figure 1.

### Characteristics of included articles

According to the risk of bias assessment, the average score of risk of bias based on 10 studies was 15.5, with all included studies demonstrating a low risk of bias. The most common items in the risk of bias assessment form associated with a higher risk of bias are related to sufficient power, and most of the included studies did not provide this information.

### Description of studies

The characteristics of the qualified studies are presented in Table 2. All retrieved research reports described studies with cross-sectional design and presented data on a total of 1000 recreational runners. There were 13 articles that estimated kinematic variables, two studies that investigated kinetic variables, two studies that explored muscle activity, and one study that evaluated spatiotemporal variables.

**TABLE 2:** Characteristics of selected studies.

Study	Sample size	Participant characteristics	Running venue condition	Variables
Ferber et al. (2003)	40 (20 females, 20 males)	Recreational runners (age 18–45 years)	Overground	Kinematic, kinetic
Takabayashi et al. (2017)	24 (12 females, 12 males)	Recreational runners (average age of female: 20.4, male: 20.7 years)	Treadmill	Spatiotemporal, kinematic
Almonroeder and Benson (2017)	32 (18 females, 14 males)	Recreational runners (average age of female: 23.7, male: 25 years)	Treadmill	Kinematic, kinetic
Willson et al. (2012)	40 (20 females, 20 males)	Varsity and recreational runners (age 18–35 years)	Overground	Kinematic, muscle activity
Vannatta and Kernozek (2018)	21	Recreational runners (average age of female: 21.9, male: 21.7 years)	Overground	Kinematic, muscle activity
Sinclair et al. (2014)	30 (15 females, 15 males)	Recreational runners (average age of female: 24.22, male: 26.98 years)	Overground	Kinematic, plantar fascia strain
Phinyomark et al. (2014)	483 (263 females, 220 males)	Recreational runners (age 18–72 years)	Treadmill	Kinematic
Rye (2017)	20 (10 females, 10 males)	Recreational runners (age 18–40 years)	Treadmill	Kinematic
Sakaguchi et al. (2014)	22 (11 females, 11 males)	Recreational runners (average age: 20.7 years)	Overground	Kinematic
Sinclair et al. (2012)	24 (12 females, 12 males)	Recreational runners (average age: 25.08 years)	Overground	Kinematic
Chumanov et al. (2008)	34 (17 females, 17 males)	Recreational runners (average age of female: 24.9 ± 4.8, male: 22.0 ± 4.8 years)	Treadmill	Kinematic
Hannigan et al. (2018)	60 (23 females, 37 males)	Experienced runners (average age of female: 29.9 ± 10.7, male: 27.4 ± 10.0 years)	Overground	Kinematic, kinetic
Schache et al. (2003)	44 (22 females, 22 males)	Recreational and elite runners (average age of female: 34.6 ± 7.3, male: 34.7 ± 6.1 years)	Treadmill	Spatiotemporal, kinematic

### Spatiotemporal variables

Ultimately, there was only one study involving spatiotemporal variables, therefore it was not possible to proceed with meta-analyses for related outcomes. Takabayashi et al. (2017)measured the speed, cadence, and step length of participants and found no differences in spatiotemporal parameters between male and female runners.

### Kinetic variables

There were only two selected studies related to kinetic parameters. Ferber, Davis, and Williams Iii (2003) investigated sex-specific differences in three-dimensional hip and knee joint moments during running and found no significant differences in knee and hip joint moments in the sagittal, frontal, or transverse planes. However, Almonroeder and Benson (2017)demonstrated that male participants showed a greater peak knee extension moment than female runners (*p* = 0.004, ES = 1.0).

### Kinematic variables

Meta-analyses were conducted for comparison of kinematic variables between male and female runners, with the exception of the kinematic parameters of the multi-segment foot due to insufficient data. Sinclair et al. (2014)investigated the sex-specific differences in kinematic variables of the multi-segment foot, as well as plantar strain, during running; the results indicated a significantly greater calcaneal eversion angle in male compared to female runners (-6.03 ± 2.33) and a larger plantar fascia strain in male versus female runners (0.09 ± 0.04). Additionally, Takabayashi et al. (2017)demonstrated a larger peak midfoot dorsiflexion angle in female compared to male runners, a significantly larger peak plantarflexion angle in the rearfoot segment of female versus male runners, and no difference between groups in the rearfoot segment in the frontal plane.

**FIGURE 2:**
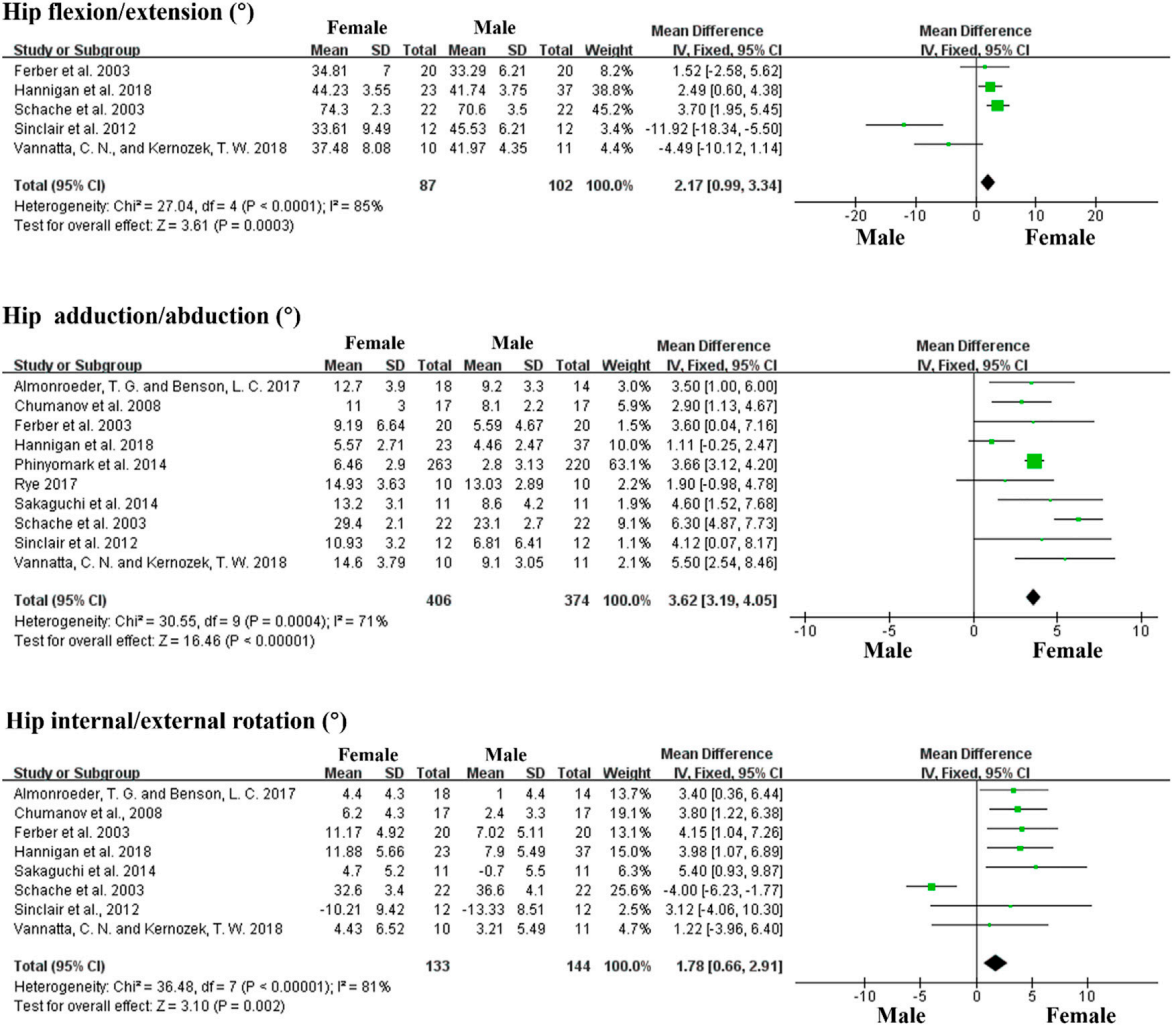
Forest plots displaying a kinematic comparison of the hip joint between female and male runners during running.

**FIGURE 3:**
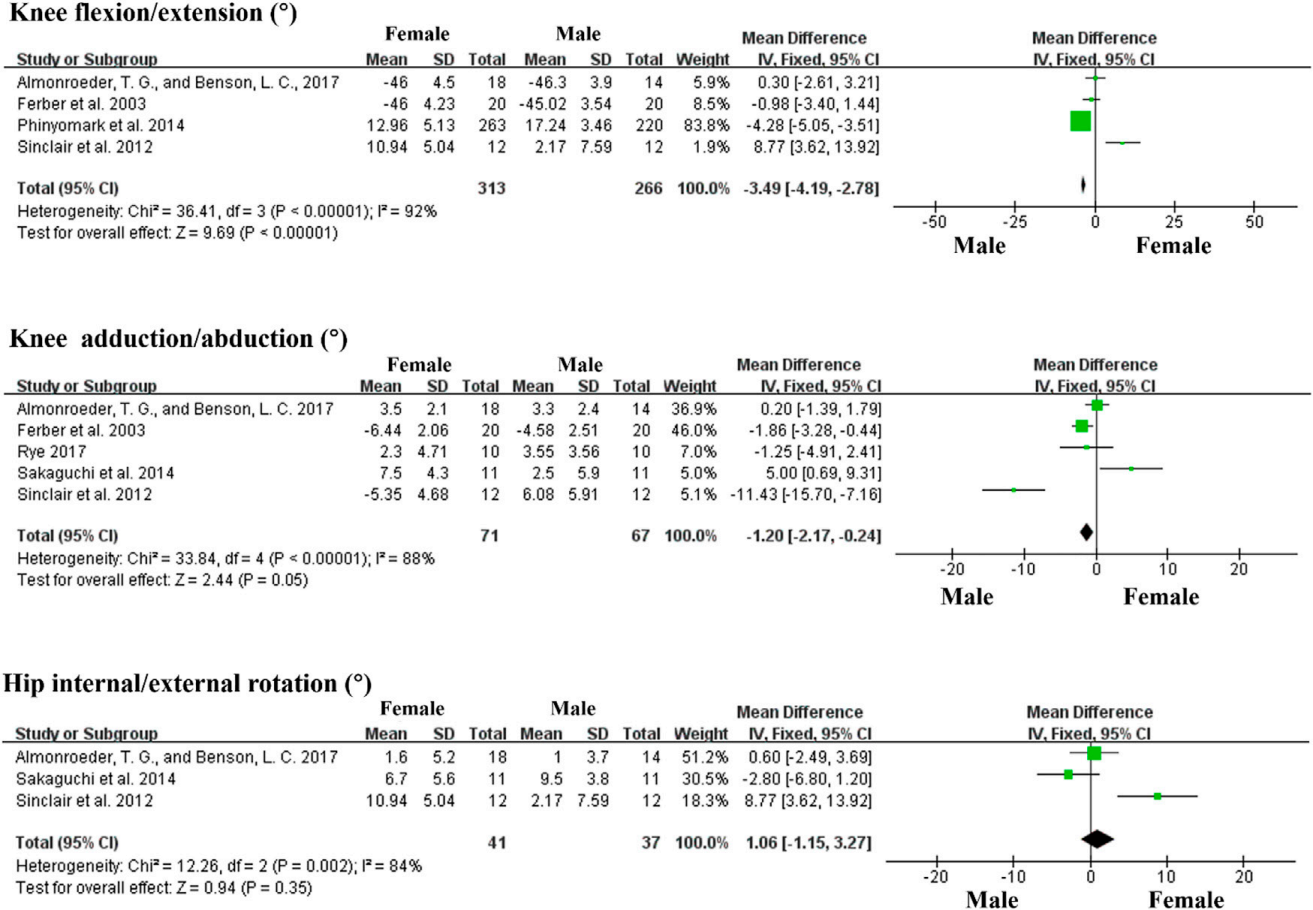
Forest plots displaying a kinematic comparison of the knee joint between female and male runners during running.

Furthermore, in terms of kinematic characteristics between female and male runners, we conducted a meta-analysis of data from 10 articles that included the following seven variables: the sagittal plane including ankle dorsiflexion/plantarflexion angle, knee flexion/extension angle, hip flexion/extension angle; the frontal plane including knee adduction/abduction angle, hip adduction/abduction angle; the transverse plane including knee internal/external rotation, hip internal rotation/external rotation angle. Data for the main kinematic variables extracted from included studies are shown in Table 3. The meta-analysis revealed that female runners demonstrated significantly greater hip flexion, adduction angle, and internal rotation angle than male runners, but smaller knee flexion angle than male runners (Forest plots are shown in Figure 2 and Figure 3).

**TABLE 3:** Kinematic parameters of included studies (“&” represents significant difference in included studies).

Study		Hip flexion/extension	Hip abduction/adduction	Hip internal/external rotation	Knee flexion/extension	Knee abduction/adduction	Knee	Ankle dorsiflexion/plantarflexion
							internal/external rotation	
Ferber et al. (2003)	Female	34.81 (7.00)	9.19 (6.64)^&^	11.17 (4.92)^&^	-46.00 (4.23)	-6.44 (2.06)^&^	-	-
	Male	33.29 (6.21)	5.59 (4.67)^&^	7.02 (5.11)^&^	-45.02 (3.54)	-4.58 (2.51)^&^	-	-
Almonroeder and Benson (2017)	Female	-	12.7 (3.9)^&^	4.4 (4.3)^&^	-45.6 (4.5)	3.5 (2.1)	1.6 (5.2)	-
	Male	-	9.2 (3.3)^&^	1.0 (4.4)^&^	−46.3 (3.9)	3.3 (2.4)	1.0 (3.7)	-
Vannatta and Kernozek (2018)	Female	37.48 (8.08)^&^	14.60 (3.79)^&^	4.43 (6.52)	-	-	-	-
	Male	41.97 (4.35)^&^	9.10 (3.05)^&^	3.21 (5.49)	-	-	--	-
Phinyomark et al. (2014)	Female	-	6.46 (2.90)^&^	-	12.96 (5.13)^&^	-	-	-21.24 (2.82)^&^
	Male	-	2.80 (3.13)^&^	-	17.24 (3.46)^&^	-	-	-24.00 (1.98)^&^
Rye (2017)	Female	-	14.93 ± 3.63	-	—	2.30 (4.71)	-	-
	Male	-	13.03 ± 2.89	-	—	3.55 (3.56)	-	-
Sakaguchi et al. (2014)	Female	-	13.2 (3.1)^&^	4.7 (5.2)^&^	-	7.5 (4.3)^&^	6.7 (5.6)	-
	Male	-	8.6 (4.2)^&^	-0.7 (5.5)^&^	-	2.5 (5.9)^&^	9.5 (3.8)	-
Sinclair et al. (2012)	Female	33.61 (9.49)^&^	10.93 (3.20)	-10.21 (9.42)	10.94 (5.04)	-5.35 (4.68)^&^	10.94 (5.04)^&^	-87.26 + 7.18
	Male	45.53 (6.21)^&^	6.81 (6.41)	-13.33 (8.51)	2.17 (7.59)	6.08 (5.91)^&^	2.17 (7.59)^&^	-86.27 + 5.75
Chumanov et al. (2008)	Female	—	11.0 (3.0)^&^	6.2 (4.3)^&^	—	—	—	—
	Male	—	8.1 (2.2)^&^	2.4 (3.3)^&^	—	—	—	—
Hannigan et al. (2018)	Female	44.23 (3.55)^&^	5.57 (2.71)	11.88 (5.66)^&^	—	—	—	—
	Male	41.73 (3.75)^&^	4.64 (2.47)	7.90 (5.49)^&^	—	—	—	—
Schache et al. (2003)	Female	74.3 (2.3)	29.4 (2.1)^&^	32.6 (3.4)	—	—	—	—
	Male	70.6 (3.5)	23.1 (2.7)^&^	36.6 (4.1)	—	—	—	—

#### Muscle activity

Willson et al. (2012) compared gluteus medius and gluteus maximus muscle activation between male and female runners and demonstrated that female runners had a larger peak gluteus maximus activation level (*p* = 0.028, effect size = 0.79) and a greater average activation level (*p* = 0.013, effect size = 0.93) compared to male runners. Additionally, Vannatta and Kernozek (2018)investigated muscle force and found that female runners had 26.9% greater hamstring force, as well as greater peak gluteus medius and gluteus minimus force, compared to male runners.

## Discussion

Results from 10 studies were used for systematic review and meta-analysis. These studies demonstrated differences in kinematic variables between female and male runners. According to the collected studies, there were no sex-specific differences in spatiotemporal or kinetic variables. Meta-analysis of the data collected for kinematic variables from 13 studies revealed that female runners exhibited significantly greater hip flexion, adduction angle, internal rotation angle, but smaller knee flexion angle, compared to male runners.

Weak lower limb muscle has been cited as a cause for increased hip adduction and internal rotation (Ireland et al., 2003; Leetun et al., 2004; Niemuth et al., 2005; Cichanowski et al., 2007; Rodrigues et al., 2020). One of the studies identified in our search revealed a significantly greater gluteus maximus muscle force in female compared to male runners. Greater gluteus maximus activation has also been reported in female runners with patellofemoral pain compared to healthy control participants (Souza and Powers 2009). It has been suggested that greater gluteus maximus activation may lead to fatigue in female runners at earlier phases of running when compared to male runners, thereby reducing force generating ability among female runners following exertion. A reduction in gluteus maximus force could disturb dynamic control of the femur and induce a kinematic adjustment following an exhaustive run (Thijs et al., 2011). Indeed, it has been demonstrated that weaker hip abductor muscles were related to increased hip adduction angle during running (Dierks et al., 2008; Dierks, Davis, and Hamill, 2010; Patra et al., 2022; Xu et al., 2022).

Kinematic differences in the hip and knee joints between male and female runners may play a prominent role in the increased incidence of injuries of the lower extremities among female runners. Previous studies have indicated that female runners have a two times higher incidence of patellofemoral pain compared to male runners (Taunton et al., 2002; Boling et al., 2010; Xiang et al., 2022). Patellofemoral pain is a result of increased stress in the patellofemoral joint, leading to a reduction in the contact area between the patella and femur as the force increases at the joint (Lee et al., 1994; Powers, 2003; Farrokhi et al., 2011; Huang and Rusanova, 2022). Our meta-analysis revealed statistically significant differences in hip and knee angles between male and female runners, with female runners demonstrating significantly greater hip internal rotation and hip adduction angles compared to male runners. Greater hip internal rotation coupled with increased knee abduction may lead to a larger dynamic quadriceps angle (Ferber, Davis, and Williams Iii, 2003). The increased dynamic quadriceps angle facilitates the lateral force acting on the patella, thereby causing dislocation of the patellofemoral joint, increasing compression of the lateral articular surface, and leading to greater lateral patellar contact force, which may contribute to a greater incidence of patellofemoral disorder (Mizuno et al., 2001) Furthermore, Ceyssens et al. (2019)summarized limited evidence from retrospective research indicating that larger peak hip adduction in female recreational runners may contribute to patellofemoral pain and iliotibial band syndrome, highlighting the important role of kinematics in the biomechanical pathology of injury.

Results from additional studies have demonstrated similar hip mechanics in female runners with patellofemoral pain. Noehren et al. (2012)compared characteristics of female runners with patellofemoral pain to those of healthy control runners; the results showed that the group with patellofemoral pain demonstrated a larger hip internal rotation and adduction than the control group. Furthermore, imaging studies have confirmed that the contact area between the patella and femur was reduced by a larger internal rotation of the femur on the patella during a single-leg squatting test, leading to greater lateral patellar displacement at the knee joint and lateral shift of pressure distribution on the patella (Li et al., 2004; Robinson and Nee, 2007). Male runners with patellofemoral pain also exhibit different running mechanics of the lower limbs compared to female runners, presenting significantly less hip adduction than female runners (Willy and Davis, 2011). Considering the sex-specific kinematic variation between female and male runners, and the possible association between patellofemoral joint stress and abnormal kinematic mechanisms, it is logical that female runners would have higher incidence of patellofemoral disorder compared with their male counterparts.

Sex-specific differences in muscle activation and kinematic variation of the lower limbs among female and male runners are important to consider when developing an individualized program for prevention of running-related injury. A potential intervention could be to provide special footwear that is designed according to the kinematic characteristics of female runners and meets specific requirements for injury protection. In addition, gluteus maximus and gluteus medius endurance training may be beneficial for adjustment of hip kinematics among female runners to improve and maintain healthy hip adduction and internal rotation. According to an 8-week rehabilitation program conducted by Earl and Hoch (2011), the patellofemoral pain extent and internal knee abduction moment reduced as hip and trunk muscle strength increased.

Additionally, comparing biomechanical characteristics of female and male runners improves our understanding of performance and running economy. Changes in running mechanics are associated with energy expenditure and, eventually, affects running performance. Previous research evaluating female runners demonstrated that a substantial decrease in VO_2_ was related to improvement of the resultant ground reaction force (GRF) and leg axis (Moore, 2016), where oxygen consumption rate is considered a determining parameter for distinguishing running performance levels (Tartaruga et al., 2012). Furthermore, several intrinsic biomechanical factors have been identified, along with related benefit to running economy, but are not exclusively associated with the lower extremities; these factors include lower vertical oscillation, small moment inertia in the leg, less leg extension at the toe-off phase, high leg stiffness, low muscle activation, etc. (Moore, 2016). In our current research, two selected studies indicated that higher muscle activation occurred in female runners compared to that of male runners, and our meta-analysis results showed greater hip flexion in female runners, which may lead to a higher vertical oscillation during running, and highlights significantly different biomechanical characteristics in female versus male runners. These differences in biomechanics may ultimately result in lower running economy among female versus male runners. In addition, Tartaruga et al. (2012)have also demonstrated a significant relationship between strike length/frequency and running economy, and a better combination of strike frequency and length could improve running economy (Dallam et al., 2005; Chen et al., 2009). More importantly, spatiotemporal characteristics are crucial elements in terms of elastic function and muscle-tendon unity during running, factors closely associated with running performance (Da Rosa et al., 2019). Unfortunately, due to the limited details provided in the selected studies, meta-analysis to compare spatiotemporal variables between female and male runners was not possible. Overall, running economy evaluation is based on the sum of influences from multiple lower-body attributes, as no single variable can completely clarify sex-specific differences in running economy (Barnes et al., 2014).

There are several limitations of our review and meta-analysis that should be considered along with our findings. First, there was significant variation in methodology among the selected articles, as methodological standardization is lacking in this field. For instance, some of the selected studies assessed the biomechanical characteristics of participants during running using a treadmill, whereas others assessed participants running overground. Differences in running biomechanics on a treadmill versus overground have been demonstrated, with treadmill running requiring less propulsion in comparison with overground running; these variations in running conditions may contribute to discrepancies in biomechanical measurements across studies (Van Hooren et al., 2020). Second, there were no requirements or restrictions related to shoe conditions, foot strike patterns, running velocity, or fatigue. Indeed, biomechanical features vary when running using different strike patterns (forefoot strike, midfoot strike, rearfoot strike), and may also be affected by running at higher speed, wearing different shoes, and fatigue status (Frishberg, 1983; García-Pérez et al., 2013; García-Pérez et al., 2014). Third, some study reports did not include all the information required for meta-analysis, therefore some data were extracted from figures, which is likely to have introduced some error.

Knowledge of sex-specific biomechanical characteristics is essential for researchers, physical therapists, and athletic trainers who work with runners. From our systematic evaluation of published data, we found no significant differences in coronal and transverse plane motion of the knee joint between male and female runners. Overall, our findings indicate that female runners demonstrate a significantly greater angle in hip flexion, hip adduction, and hip internal rotation, but a smaller knee flexion angle when compared to male runners. These differences likely increase patellofemoral joint stress and thus increase the risk of patellofemoral joint disorder in female runners. Therefore, sex-specific differences are important and should be considered in footwear design and in development of individualized injury prevention programs.

## Data Availability

The raw data supporting the conclusion of this article will be made available by the authors, without undue reservation.

## Appendix A1 Methods of risk of bias assessment.

**Table:**

Criteria	Description
1. Aim clearly described	The aim/hypothesis/objective is clearly described.
2. Outcomes described	The main outcomes to be measured are clearly described in the introduction or methods section.
3. Subjects clearly described	The characteristics of the subjects included in the trial are clearly described. If running experience was deemed insufficiently described, this was answered no.
4. Interventions clearly described	Each intervention to be compared is clearly described.
5. Distribution of confounders described	Confounding factors are clearly described. Confounders to be considered include subject’s sex, age, weight, running experience, running speed, and foot strike.
6. Main findings clearly described	Simple outcome data are reported for all major findings so the reader can check the major analyses and conclusions.
7. Estimates of random variability in data	In non-normally distributed data, the interquartile range of results should be reported. In normally distributed data, standard deviations or confidence intervals should be reported.
8. All important adverse events reported	The study demonstrates a comprehensive attempt to record all adverse events. This could include discomfort associated with any running condition or delayed-onset muscle soreness.
9. Actual probability values reported	Actual probability values (e.g., not *p* <.05) have been reported for the main outcomes, except where the probability value is less than .001.
10. Subjects asked are representative of population	The source population for subjects and how they were selected are described. Subjects would be representative if they comprised the entire population, an unselected sample of consecutive subjects, or a random sample. Where the study does not report the proportion of the source population from which the subjects are derived, the answer is no.
11. Subjects representative of population	The subjects prepared to participate are representative of the entire population from which they were recruited. Validation that the sample was representative would include demonstrating that the distribution of confounding factors was the same in the study sample and the source population.
12. Examiners blinded	There was an attempt to blind those measuring the main outcomes.
13. Data dredging	Any analysis that had not been planned at the outset of the study is clearly described. If no retrospective unplanned subgroup analysis is reported, the answer is yes.
14. Appropriate statistical tests	The statistical tests used to assess the main outcomes are appropriate.
15. Valid and reliable main outcome measures	For studies where the outcome measures are clearly described, the answer is yes. For studies that refer to other work or that demonstrate the outcome measures are accurate, the answer is yes.
16. Subjects recruited from same population	The subjects in different intervention groups were recruited from the same population. If the subjects acted as their own control, this was answered yes.
17. Subjects recruited over same time period	The subjects in different intervention groups were recruited over the same time period. If the subjects acted as their own control, this was answered yes.
18. Intervention order randomized	The order of the intervention tested was randomized.
19. Adequate adjustments for confounding	There was adequate adjustment for confounding in the analysis from which the main findings were drawn. If the effect of the main confounders was not investigated or confounding was demonstrated but no adjustment was made in the final analysis, this was answered no.
20. Sufficient power	If the study reported a power calculation, this was answered yes.
